# Safety and Efficacy of a Sandwich Total Neoadjuvant Therapy Strategy for Low‐Risk Distal Locally Advanced Rectal Cancer: Results From the TESS Phase II Trial

**DOI:** 10.1002/mco2.70807

**Published:** 2026-06-17

**Authors:** Shuang Liu, GuangZhao Lv, GeYu Xu, XiaoZhong Wang, YeZhong Zhuang, ShouMin Bai, HaiNa Yu, XiaoJun Wu, YiJing Ye, HuiLong Luo, ShuoYu Xu, QiaoXuan Wang, Hui Chang, PeiQiang Cai, ZhiZhong Pan, YuanHong Gao, Gong Chen, WeiWei Xiao

**Affiliations:** ^1^ Department of Radiation Oncology Sun Yat‐Sen University Cancer Center Guangzhou China; ^2^ State Key Laboratory of Oncology in South China Collaborative Innovation Center For Cancer Medicine Sun Yat‐Sen University Cancer Center Guangzhou China; ^3^ Department of Hematology Oncology, and Tumor Immunology and Berlin Institute of Health Charité‐Universitätsmedizin Berlin Berlin Germany; ^4^ Berlin Institute for Medical Systems Biology Max Delbrück Center for Molecular Medicine Berlin Germany; ^5^ Department of Colorectal Surgery Sun Yat‐Sen University Cancer Center Guangzhou China; ^6^ Department of Medical Imaging and Interventional Radiology Sun Yat‐Sen University Cancer Center Guangzhou China; ^7^ Department of General Surgery Shantou Central Hospital Shantou China; ^8^ Department of Abdominal Surgery Cancer Hospital of Shantou University Medical College Shantou China; ^9^ Department of Radiation Oncology Sun Yat‐Sen Memorial Hospital Sun Yat‐Sen University Guangzhou China; ^10^ Department of Medical Oncology Jinjiang Municipal Hospital (Shanghai Sixth People's Hospital Fujian) Jinjiang China; ^11^ Department of Radiation Oncology Zhongshan People's Hospital Zhongshan China; ^12^ Bio‐Totem Pte Ltd Suzhou China; ^13^ United Laboratory of Frontier Radiotherapy Technology of Sun Yat‐Sen University & Chinese Academy of Sciences Ion Medical Technology Co., Ltd Guangzhou China

**Keywords:** clinical complete response, rectal cancer, sphincter preservation, total neoadjuvant treatment, watch‐and‐wait strategy

## Abstract

This prospective phase II multicenter study evaluated a novel sandwich total neoadjuvant treatment (TNT) strategy for patients with relatively low‐risk distal locally advanced rectal cancer (LARC). The regimen consisted of two cycles of capecitabine and oxaliplatin (CapeOx) administrated before, during, and after radiotherapy, followed by surgery or a watch‐and‐wait strategy, and two cycles of adjuvant capecitabine chemotherapy. The primary endpoint was the clinical complete response (cCR) rate. All 98 patients completed radiotherapy, and 88.8% received six cycles of neoadjuvant chemotherapy. In the intention‐to‐treat analysis, 46.9% of patients achieved cCR, 7.1% achieved near‐clinical complete response (near‐cCR), and 45.9% had non‐clinical complete response (non‐cCR). In the observed population, the complete response (CR), pathological complete response (pCR), and sphincter‐preservation (SP) rates were 61.2%, 46.4%, and 79.6%, respectively. The local regrowth‐free survival, local recurrence‐free survival, distant metastases‐free survival, and overall survival rates were 89.5%, 100%, 92.9%, and 96.9%, respectively. Overall, this sandwich TNT strategy achieved high rates of cCR, pCR, CR, and SP, with favorable survival outcomes and acceptable toxicity, suggesting a promising organ‐preserving treatment approach for selected patients with distal LARC.

Trial Registration: ClinicalTrials.gov identifier: NCT03840239

## Graphical abstract

The proposed sandwich total neoadjuvant treatment (TNT) strategy (NeoCT–CRT–NeoCT) represents a safe and effective therapeutic option for carefully selected patients with low‐risk distal locally advanced rectal cancer (LARC). By sequentially integrating induction chemotherapy, concurrent chemoradiotherapy, and consolidation chemotherapy, this approach significantly improves clinical complete response (cCR) rates, facilitates sphincter preservation, and optimizes both oncological outcomes and long‐term quality of life.

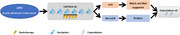

## 1 Introduction

Neoadjuvant chemoradiotherapy (CRT) followed by total mesorectal excision (TME), with or without adjuvant chemotherapy, has long been the standard treatment for patients with locally advanced rectal cancer (LARC). This treatment paradigm has substantially improved locoregional control, reducing local recurrence rates to below 10%. However, distant metastasis remains a major cause of treatment failure, and the survival benefit of postoperative adjuvant chemotherapy remains uncertain despite extensive investigation over the past decade [1, 2, 3]. Furthermore, several studies have questioned the clinical value of adjuvant chemotherapy after CRT and TME in LARC patients [4, 5, 6].

To address these limitations, total neoadjuvant treatment (TNT) strategies, which deliver systemic chemotherapy and radiation before surgery, have increasingly been explored. A study from Memorial Sloan‐Kettering Cancer Center demonstrated that TNT could improve chemotherapy compliance and tumor response compared with conventional CRT followed by adjuvant chemotherapy [7]. Subsequently, multiple prospective clinical trials have confirmed that TNT strategies can achieve higher pathological complete response (pCR) rates, better treatment compliance, and potentially improved survival outcomes [8, 9, 10, 11, 12]. However, most large randomized TNT trials, including RAPIDO, PRODIGE‐23, and STELLAR, have primarily focused on pathological response and survival endpoints, with limited emphasis on clinical complete response (cCR) and organ preservation as primary outcomes [8, 9, 10, 12].

For patients with distal LARC, organ preservation is an important therapeutic goal. Radical surgical procedures such as abdominoperineal resection may lead to permanent colostomy and significantly impair patients’ quality of life and functional outcomes. In this context, both sphincter‐preserving surgery (SPS) and the watch‐and‐wait (W&W) strategy have emerged as promising alternatives for patients who achieve cCR after neoadjuvant therapy. Evidence from several studies has shown that the W&W approach can achieve oncological outcomes comparable to those of TME while preserving organ function and improving quality of life [13, 14, 15]. Therefore, optimizing neoadjuvant treatment strategies to maximize the probability of achieving cCR has become an important research priority in distal rectal cancer management.

Among existing TNT paradigms, two main treatment sequences are widely adopted: induction chemotherapy followed by CRT (NeoCT–CRT) and CRT followed by consolidation chemotherapy (CRT–NeoCT). Previous studies have suggested that the consolidation strategy may lead to greater tumor regression and higher pCR rates [16]. However, prolonged intervals between radiotherapy and surgery may increase pelvic fibrosis, potentially complicating surgical procedures and affecting the completeness of mesorectal excision [17, 18]. Therefore, treatment strategies that maintain the tumor‐regression advantages of consolidation chemotherapy while minimizing these surgical challenges warrant investigation.

In addition, the role of oxaliplatin in concurrent CRT remains controversial. Some clinical trials have reported that adding oxaliplatin to fluorouracil‐based CRT increased toxicity without improving tumor regression [19, 20, 21]. However, other studies have demonstrated that the combination of oxaliplatin and fluoropyrimidines with long‐course radiotherapy can achieve acceptable toxicity profiles and improved tumor responses compared with fluoropyrimidine‐based CRT alone [22, 23, 24, 25, 26, 27, 28].

Based on these considerations, we developed a novel “sandwich” TNT strategy (NeoCT–CRT–NeoCT), in which two cycles of capecitabine and oxaliplatin (CapeOx) are administered before and after long‐course CRT. This approach sequentially employs induction chemotherapy to reduce tumor burden and enhance radiosensitivity, concurrent CRT for local synergy, and consolidation chemotherapy to eliminate residual disease, all within a compact sequence that shortens the interval between radiotherapy and surgery. Importantly, this strategy was designed for patients with low‐risk distal LARC, a subgroup in which achieving cCR and enabling organ preservation are particularly clinically meaningful.

In this prospective multicenter phase II study (the TESS trial; TNT to increase the cCR rate for distal LARC), we evaluated the safety and efficacy of this sandwich strategy. The primary endpoint was the cCR rate, reflecting the study's focus on organ preservation. In addition, we conducted an exploratory analysis to investigate whether artificial intelligence‐based analysis of pretreatment whole‐slide histopathology images could identify histopathological features associated with treatment response [29, 30, 31, 32, 33, 34].

## 2 Results

### 2.1 Study Population

Between February 2019 and December 2021, 98 patients with LARC were enrolled from across five participating centers. The mean patient age was 55.1 years (range, 45.3–64.9 years). All tumors were located within 5 cm of the anal verge, with a median distance of 3.4 cm (interquartile range [IQR] 2.8–4.3 cm). Baseline clinical characteristics are summarized in Table 1. Most patients had cT3 disease (82.7%), while 11.2% had cT4a tumors. Lymph node involvement was present in 71.4% of patients. Mesorectal fascia involvement was observed in 45.9%, and extramural venous invasion was detected in 23.5% of cases.

**TABLE 1:** Baseline demographic and clinical characteristics of patients with LARC (*N* = 98).

Characteristics	*N* (%)
Age	
Mean ± SD	55.1 ± 9.8
<55	44 (44.9%)
≥55	54 (55.1%)
Sex	
Female	34 (34.7%)
Male	64 (65.3%)
Distance to the anal verge (cm)	
Median (IQR)	3.4 (2.8‒4.3)
<3.5	50 (51.0%)
≥3.5	48 (49.0%)
Center	
Sun Yat‐Sen University Cancer Center	86 (87.8%)
Shantou Central Hospital	5 (5.1%)
Cancer Hospital of Shantou University Medical College	4 (4.1%)
Sun Yat‐Sen Memorial Hospital	2 (2.0%)
Zhongshan People's Hospital	1 (1.0%)
T stage	
T2	6 (6.1%)
T3	81 (82.7%)
T4a	11 (11.2%)
N stage	
N0	28 (28.6%)
N1	43 (43.9%)
N2	27 (27.6%)
MRF	
Negative	53 (54.1%)
Positive	45 (45.9%)
EMVI	
Negative	75 (76.5%)
Positive	23 (23.5%)

Abbreviations: EMVI, extramural venous invasion; IQR, interquartile range; MRF, mesorectal fascia; SD, standard deviation.

### 2.2 Treatment Administration and Compliance

Treatment details are summarized in Table 2. All 98 patients (100%) completed the planned course of radiotherapy. The majority of patients (88/98, 88.8%) completed six cycles of neoadjuvant chemotherapy, indicating high treatment compliance. Eight patients (8.2%) received a second course of radiotherapy (30–40 Gy in 15–20 fractions). Among these patients, seven were initially classified as non‐clinical complete response (non‐cCR) and one as near‐clinical complete response (near‐cCR) following the TNT regimen. After the second radiotherapy course, four patients achieved cCR and subsequently avoided surgery, while the remaining four patients underwent TME, including three non‐SPSs and one SPS. Detailed information on the patients who received a second course of radiotherapy is provided in Figure S1.

**TABLE 2:** Treatment administration and compliance in the study cohort (*N* = 98).

Characteristics	*N* (%)
Cycles of neoadjuvant chemotherapy	
4	4 (4.1%)
5	7 (7.1%)
6	80 (81.6%)
7	5 (5.1%)
8	2 (2.0%)
Radiotherapy	
First course of radiotherapy completed	98 (100%)
Total dose (Gy)	50
Fractions	25
Second course of radiotherapy^a^	8 (8.2%)
Dose of second course (Gy)	30‒40
Fractions of second course	15‒20
Surgical management	
Sphincter‐preservation surgery	40 (40.8%)
Non‐sphincter‐preservation surgery	16 (16.3%)
Declined surgery	11 (11.2%)
Watch‐and‐wait	31 (31.6%)
Cycles of adjuvant chemotherapy	
2	95 (96.9%)
3	1 (1.0%)
4	2 (2.0%)

^a^ Details of the eight patients who received a second course of radiotherapy are shown in Figure S1.

### 2.3 Tumor Response and Clinical Outcomes

**FIGURE 1:**
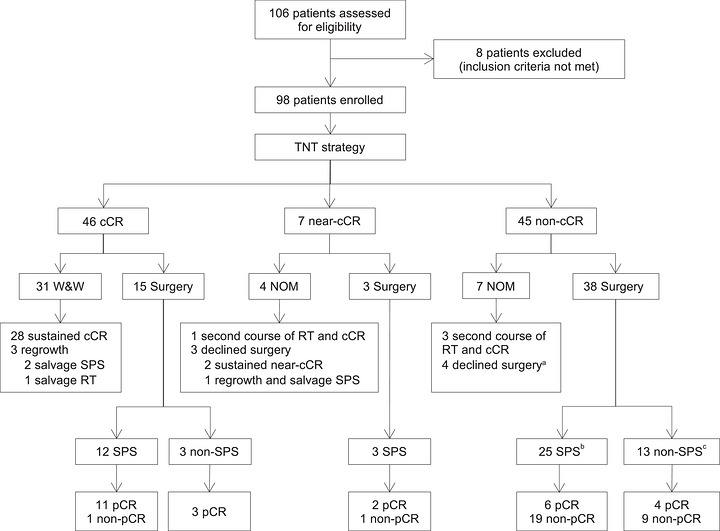
Flow diagram and initial response, including watch‐and‐wait (W&W) and surgery, in the intention‐to‐treat population. ^a^One patient was diagnosed with tumor regrowth 34.7 months after the end of treatment and received chemotherapy. One patient developed metastasis and died. Two patients were not reviewed regularly and did not complain of any obvious discomfort. ^b^One patient received a second course of radiotherapy (RT) before surgery, and the surgical pathology result was non‐pathological complete response (non‐pCR). ^c^Three patients received a second course of RT before surgery. Among them, one achieved pCR and two had non‐pCR on surgical pathology. cCR, clinical complete response; near‐cCR, near‐clinical complete response; NOM, non‐operative management; non‐cCR, non‐clinical complete response; non‐SPS, non‐sphincter‐preservation surgery; SPS, sphincter‐preservation surgery; TNT, total neoadjuvant treatment.

Figure 1 illustrates the initial treatment response and subsequent management strategies, including W&W and surgical treatment. Among the 98 patients, 46 (46.9%) achieved cCR, seven (7.1%) achieved near‐cCR, and 45 (45.9%) were classified as non‐cCR.

**FIGURE 2:**
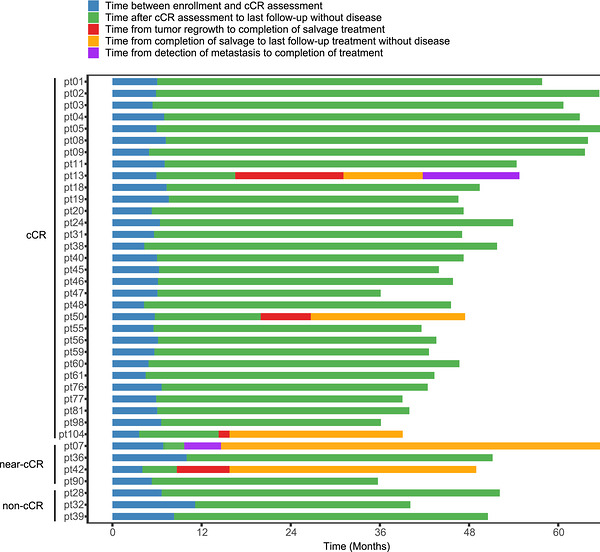
Follow‐up of patients who received the watch‐and‐wait (W&W) strategy. Four patients (pt07, pt36, pt42, and pt90) were evaluated as near‐clinical complete response (near‐cCR), and three patients (pt28, pt32, and pt39) were evaluated as non‐clinical complete response (non‐cCR) at the first assessment. Four patients (pt28, pt32, pt36, and pt39) received a second course of radiotherapy and achieved clinical complete response (cCR). Further details of these patients who underwent a second course of radiotherapy are also provided in Figure S1. Patients assessed as cCR or near‐cCR at initial total neoadjuvant treatment (TNT) evaluation were included in the ITT W&W population. Those initially classified as non‐cCR who achieved cCR after a second course of radiotherapy were excluded from ITT W&W population but included in the actual W&W population.

Among the 46 patients with cCR, 31 (67.4%) adopted the recommended W&W strategy. During follow‐up, three patients experienced tumor regrowth, occurring 10.5, 10.75, and 15 months after the initial cCR assessment. Two of these patients underwent salvage SPS, while one received salvage radiotherapy because surgery was contraindicated. Follow‐up details of patients managed with W&W are shown in Figure 2. The remaining 15 patients with cCR (32.6%) underwent TME. Among them, 14 (93.3%) achieved pCR, while one did not achieve pCR (non‐pCR).

After completion of the TNT regimen, seven patients (7.1%) achieved near‐cCR. Three of these patients underwent salvage SPS, including two with pCR and one with non‐pCR. Of the remaining four patients managed non‐operatively, one received a second course of radiotherapy, subsequently achieved cCR, and adopted the W&W strategy. One patient developed tumor regrowth 4.5 months after cCR assessment and underwent salvage SPS, while the remaining two patients remained free from tumor regrowth during follow‐up.

After completion of the TNT regimen, 45 patients (45.9%) were classified as non‐cCR. Among these patients, 25 underwent SPS (including six pCR and 19 non‐pCR cases) and 13 underwent non‐SPS (including four pCR and nine non‐pCR cases). Notably, three patients achieved cCR after receiving a second course of radiotherapy and subsequently adopted W&W, while four patients declined surgical intervention.

Overall, 56 patients underwent surgery, including 40 patients (71.4%) who received SPS and 16 patients (28.6%) who underwent non‐SPS. The overall pCR rate among surgical patients was 46.4% (26/56). Surgery was performed at a median of 5.7 weeks (IQR 3.8–8.5) after completion of CRT and 11.7 weeks (IQR 9.8–14.5) after the final fraction of radiotherapy. Seven patients experienced delayed surgery due to the COVID‐19 pandemic. Additional surgical procedures and postoperative complications are summarized in Table 3.

**TABLE 3:** Surgical outcomes and postoperative complications (*N* = 56).

Characteristics	Value
Surgery performed, *N* (%)	
Sphincter‐preserving surgery	40 (71.4%)
Non‐sphincter‐preserving surgery	16 (28.6%)
Postoperative hospital stay (days)	
Median (IQR)	6 (5–8)
Duration of surgery (min)	
Median (IQR)	278 (239–311)
Estimated blood loss (mL)	
Median (IQR)	50 (30–50)
Time from end of RT to surgery (weeks)^a^	
Median (IQR)	11.7 (9.8–14.5)
Time from end of CRT to surgery (weeks)^b^	
Median (IQR)	5.7 (3.8–8.5)
Delayed surgery due to COVID‐19 pandemic	7
ypT classification, *N* (%)	
T0	26 (46.4%)
T1	1 (1.8%)
T2	13 (23.2%)
T3	15 (26.8%)
T4a	1 (1.8%)
ypN classification, *N* (%)	
N0	52 (92.9%)
N1	2 (3.6%)
N2	2 (3.6%)
Surgical complications, *N* (%)	
Anastomotic leakage	2 (3.6%)
Rectovaginal fistula	1 (1.8%)

Abbreviations: CRT, chemoradiotherapy; IQR, interquartile range; RT, radiotherapy.

^a^ Patients who received a second course of radiotherapy followed by surgery were excluded from this analysis; details are provided in Figure S1.

^b^ Includes all patients who underwent surgery.

For the intention‐to‐treat (ITT) analysis, the overall complete response (CR) rate was 58.2% (57/98). This included 26 patients who achieved pCR following surgery, as well as 31 patients managed with W&W with sustained response for at least 1.5 years, including 28 with sustained cCR, two with sustained near‐cCR, and one who converted from near‐cCR to cCR following a second course of radiotherapy. The overall SP rate was 75.5% (74/98). This included 43 patients who underwent SPS (including three salvage SPS procedures, but excluding one who underwent SPS following a second course of radiotherapy), the aforementioned 31 W&W patients, and one who received salvage radiotherapy with tumor regrowth after cCR assessment. In the observed response analysis, the CR rate was 61.2% (60/98) and the SP rate was 79.6% (78/98). Three patients who achieved cCR after a second course of radiotherapy (initially classified as non‐cCR) were excluded from the ITT CR and SP analyses but included in the observed response analysis. Additionally, one patient who underwent SPS following a second course of radiotherapy was excluded from the ITT SP analysis but included in the observed SP analysis.

### 2.4 Adverse Events

Table 4 summarizes the adverse event profile. Grade ≥3 hematological toxicities occurred in 27 out of 98 patients (27.6%), with thrombocytopenia being the most common severe hematological toxicity (12.3%, 12/98). Grade 3 radiation enteritis was observed in two patients (2.0%), and grade 3 hand–foot syndrome occurred in one patient (1.0%). Grade ≥3 gastrointestinal toxicities were reported in five patients (5.1%), primarily due to diarrhea (5/98, 5.1%). One patient (1.0%) experienced grade 3 abdominal pain.

**TABLE 4:** Adverse events.

Grade	Grade 0	Grade 1	Grade 2	Grade 3	Grade 4
Hematological toxicities	11 (11.2%)	27 (27.6%)	33 (33.7%)	24 (24.5%)	3 (3.1%)
Thrombocytopenia	45 (45.9%)	22 (22.4%)	19 (19.4%)	9 (9.2%)	3 (3.1%)
Anemia	39 (39.8%)	39 (39.8%)	14 (14.3%)	6 (6.1%)	0
Leukopenia	29 (29.6%)	26 (26.5%)	35 (35.7%)	8 (8.2%)	0
Neutropenia	38 (38.8%)	30 (30.6%)	18 (18.4%)	12 (12.2%)	0
Hepatic dysfunction	21 (21.4%)	64 (65.3%)	12 (12.2%)	1 (1%)	0
Radiation enteritis	85 (86.7%)	3 (3.1%)	8 (8.2%)	2 (2%)	0
Neurological toxicity	89 (88.8%)	7 (7.1%)	2 (2%)	0	0
Hand–foot syndrome	97 (99%)	0	0	1 (1%)	0
Gastrointestinal toxicities	60 (61.2%)	21 (21.4%)	12 (12.2%)	5 (5.1%)	0
Diarrhea	78 (79.6%)	5 (5.1%)	10 (10.2%)	5 (5.1%)	0
Abdominal pain	88 (89.8%)	8 (8.2%)	1 (1%)	1 (1%)	0
Proctitis	92 (93.9%)	4 (4.1%)	2 (2%)	0	0
Rectal pain	94 (95.9%)	4 (4.1%)	0	0	0
Nausea	92 (93.9%)	6 (6.1%)	0	0	0
Vomiting	92 (93.9%)	6 (6.1%)	0	0	0

### 2.5 Survival Outcomes

**FIGURE 3:**
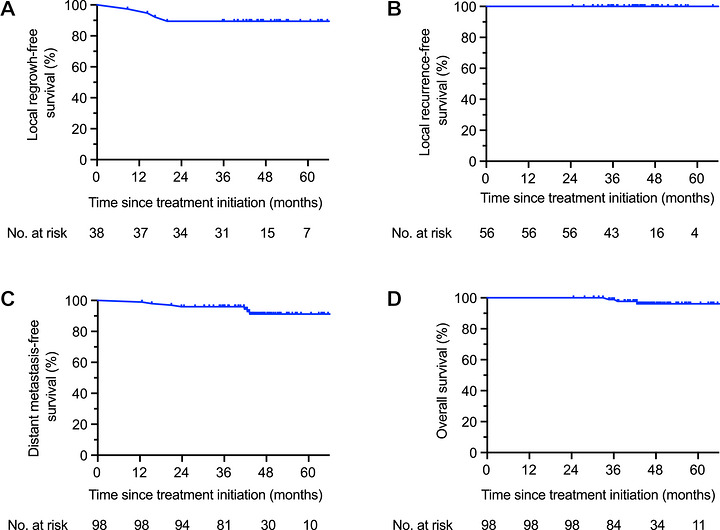
Local regrowth‐free survival, local recurrence‐free survival, distant metastasis‐free survival, and overall survival. Kaplan‒Meier estimates of (A) local regrowth‐free survival in the actual watch‐and‐wait population, (B) local recurrence‐free survival, (C) distant metastasis‐free survival, and (D) overall survival.

During the W&W strategy, four of 38 patients (10.5%) experienced local regrowth, corresponding to a local regrowth‐free survival rate of 89.5% (Figure 3A). Among the 56 patients who underwent surgery, no cases of local recurrence were observed, resulting in a local recurrence‐free survival rate of 100% (Figure 3B). Seven patients developed distant metastases, corresponding to a distant metastasis‐free survival rate of 92.9% (Figure 3C). After a median follow‐up of 44.4 months (IQR 40.2–51.5), three patients had died, yielding an overall survival rate of 96.9% (Figure 3D). The final follow‐up was completed in November 2024.

### 2.6 Subgroup Analysis: CR Versus Non‐Complete Response (Non‐CR) Patients

Baseline clinical characteristics and treatment parameters of CR and non‐CR patients in the ITT population are summarized in Table S1, while the corresponding analyses based on observed response are presented in Table S2. Three patients who achieved cCR after a second course of radiotherapy (initially classified as non‐cCR) were excluded from the ITT CR group but included in the observed response analysis. No significant differences in baseline characteristics or treatment parameters were observed between the two groups.

### 2.7 Exploratory Histopathological Analysis

We evaluated the associations between pretreatment histopathologic features and tumor response using H&E‐stained whole‐slide images (WSIs) from 50 patients (30 CR and 20 non‐CR). Baseline characteristics and treatment parameters for this cohort are summarized in Table S3, and the results of the univariate analyses are presented in Table S4.

**FIGURE 4:**
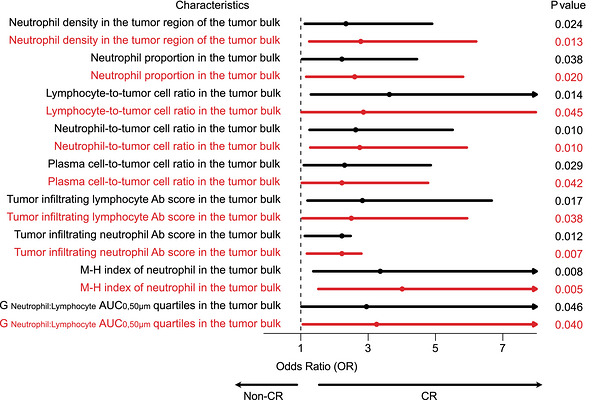
Exploratory histopathological analysis comparing non‐complete response (non‐CR) and complete response (CR) patients. Univariate (black) and multivariate (red) logistic regression analyses for tumor response. The multivariable (red) analysis initially included sex, age, clinical T stage, tumor distance to the anal verge, and the cycles of neoadjuvant chemotherapy. Ab, abundance; M‒H index, Morisita‒Horn index. Data were log‐transformed to improve normality.

Regarding immune cell density and proportion, univariate and multivariable logistic regression analyses (Figures 4 and S2 and Table S4) showed that higher neutrophil abundance (Ab) was significantly associated with improved tumor response. In contrast, lymphocyte proportion, but not lymphocyte density, was associated with better treatment response.

Analysis of immune cell spatial distribution (Figures 4 and S3 and Table S4) showed that both the neutrophil Ab score and the Morisita–Horn (M–H) index, which reflects immune–tumor cell colocalization, were significantly associated with treatment response in both univariate and multivariable models. In addition, lymphocyte and neutrophil Ab scores demonstrated significant associations with tumor response.

Finally, analysis of cell–cell interaction metrics revealed that a higher *G*‐cross value (*G*_Lymphocyte:Neutrophil_ AUC_0,50 µm_), representing the probability of lymphocyte–neutrophil proximity within 0–50 µm, was associated with improved response (odds ratio [OR] 3.25, 95% CI 1.06–9.98; *p* = 0.04).

## 3 Discussion

This study demonstrates that, in LARC patients, a novel sandwich TNT strategy (NeoCT‒CRT‒NeoCT), consisting of two cycles of CapeOx administered before, during, and after long‐course radiotherapy, followed by TME or W&W and adjuvant chemotherapy, achieves high rates of cCR, pCR, and overall SP. The regimen also demonstrated favorable treatment compliance, encouraging survival outcomes, and manageable toxicity. Notably, the W&W strategy proved to be a clinically feasible and oncologically safe option for patients achieving cCR after neoadjuvant therapy. In addition, exploratory histopathological analysis of pretreatment H&E‐stained WSIs suggested that the Ab and spatial distribution of neutrophils and lymphocytes within the tumor microenvironment may be associated with improved tumor response in LARC.

Over the past decade, multiple trials exploring various TNT strategies have consistently shown improvements in pCR rates in LARC patients. The PRODIGE‐23 trial evaluated a TNT regimen consisting of FOLFIRINOX followed by long‐course concurrent CRT (50.4 Gy in 25 fractions with capecitabine), reporting a significantly higher pCR rate in the TNT group compared with standard treatment (28% vs. 12%). Similarly, the CAO/ARO/AIO‐12 trial compared induction and consolidation TNT strategies using fluorouracil–oxaliplatin chemotherapy with long‐course radiotherapy, demonstrating higher pCR rates in the consolidation arm (28% vs. 14%; *p* < 0.0001) [25, 26, 35]. The RAPIDO and STELLAR trials evaluated short‐course radiotherapy followed by chemotherapy (RAPIDO: CAPOX or FOLFOX; STELLAR: CapeOx) [12, 36, 37]. RAPIDO reported a doubling of the pCR rate in the TNT arm (28% vs. 14%; *p* < 0.0001), while STELLAR demonstrated that the combined pCR and sustained cCR rate was significantly higher than in the standard‐treatment group (21.8% vs. 12.3%; *p* = 0.002). However, most of these trials focused primarily on pathological response and survival endpoints, with limited emphasis on SP and organ function, thereby restricting the translation of pCR improvements into functional outcomes.

The OPRA trial specifically addressed organ preservation, using TME‐free survival as a key outcome [38, 39]. In that study, patients with clinical T3‐4N0/TanyN1‐2 disease received induction or consolidation TNT. Tumor location in OPRA was generally higher than in our cohort (median distance from the anal verge: induction arm 4.3 cm [IQR 3.0–6.3 cm] and consolidation arm 4.5 cm [IQR 3.0–6.5 cm], compared with 3.0 cm [IQR 2.8–4.3 cm] in the present study).

The cCR/near‐cCR rate in OPRA (71% and 76% in the induction and consolidation arms, respectively) was higher than the ITT cCR (46.9%) and near‐cCR (7.1%) rates observed in our study. Nevertheless, the local regrowth rate among W&W patients in our cohort was only 10.5%, substantially lower than that reported in OPRA (40% in the induction arm and 27% in the consolidation arm) and in other studies [40, 41, 42]. In addition, surgical patients in our cohort demonstrated an overall pCR rate of 46.6%, with pCR rates of 93.3%, 66.7%, and 26.3% in the cCR, near‐cCR, and non‐cCR subgroups, respectively. These values are notably higher than those reported in OPRA (pCR rates of 100%, 15.8%, and 7.8% in the corresponding subgroups). These differences may reflect more stringent criteria for defining cCR and near‐cCR in our study, which may be particularly important for avoiding inappropriate recommendations for non‐operative management.

Accurate assessment of cCR is essential for the safe implementation of W&W. In our study, pelvic magnetic resonance imaging (MRI) was used in 100% of cases, compared with approximately 71% in the International Watch & Wait Database registry studies. Comprehensive evaluation combining MRI, endoscopy, and digital rectal examination likely improves diagnostic accuracy and facilitates the early detection of regrowth. In our previous propensity‐matched study, the regrowth rate among W&W patients after neoadjuvant CRT was 12%, which is consistent with the findings of the present study [14].

Notably, 32.6% of patients who achieved cCR, still opted for surgical intervention. During the shared decision‐making process, patients were thoroughly informed about the potential risks and benefits of W&W. Some patients expressed concerns regarding the possibility of tumor regrowth, which influenced their decision to pursue surgical management. Despite these concerns, the regrowth rate among patients who adopted W&W after rigorous evaluation remained remarkably low (10.5%).

Looking forward, broader adoption of the W&W strategy for eligible cCR patients may be facilitated by improved patient education and clinician communication. Strengthening confidence in W&W among both patients and physicians may help maximize the potential benefits of this approach in appropriately selected individuals.

In our study, eight patients received a second course of radiotherapy as part of an organ‐preservation strategy (Figure S1). This approach is supported by our previous work demonstrating sustained cCR and favorable disease‐free survival following dose escalation [43]. Similarly, the OPERA trial showed that increasing the dose using contact X‐ray brachytherapy improved the organ‐preservation rate with favorable bowel function at 3 years [44]. The WW2 study further demonstrated that 61% of patients with low rectal cancer could be successfully treated with 62 Gy delivered in 28 fractions, achieving excellent patient‐reported outcomes, tumor control, and survival [45, 46]. Nevertheless, the optimal radiation dose for the second‐course radiotherapy and the risk of delayed hemorrhage associated with high radiation doses require further investigation.

Recent studies have also explored combining CRT with immunotherapy, which can increase cCR rates but may introduce immune‐related adverse events, including thyroid dysfunction and colitis, some of which may be lifelong [47, 48, 49]. In this context, our sandwich TNT strategy represents a cost‐effective approach with manageable toxicity, potentially offering an alternative for patients who are not candidates for immunotherapy.

The survival outcomes observed in our cohort were also encouraging and appeared superior to those reported in several historical TNT studies (Table S5) [8, 9, 10, 15]. This may partly reflect the selection of relatively low‐risk patients, as individuals with T4b disease or lateral lymph node metastasis were excluded. In addition, the cohort was enriched with distal tumors (<5 cm from the anal verge), which are particularly relevant for organ‐preserving approaches. The compact sandwich TNT sequence, which integrates dual‐agent chemotherapy before, during, and after radiotherapy, may also contribute to improved treatment efficacy, although the single‐arm design of this study limits causal inference.

Toxicity associated with oxaliplatin‐based concurrent CRT was acceptable and consistent with previous studies [22, 23, 24]. In our cohort, grade ≥3 hematologic toxicities occurred in 27.6% of patients, with thrombocytopenia occurring in 12.3%, and hand‒foot syndrome in 1.0%. These rates are comparable to those reported in the TNTCRT trial (grade ≥3 thrombocytopenia 10.34%; grade ≥3 hand–foot syndrome 2.59%) [50]. In addition, the FOWARC trial demonstrated that full‐dose fluorouracil‒oxaliplatin combined with radiotherapy significantly improved pCR rates compared with fluorouracil alone (27.5% vs. 14.0%), supporting the use of this regimen in patients requiring aggressive tumor downstaging.

The tumor immune microenvironment may also play an important role in treatment response. High lymphocyte infiltration has been widely recognized as a favorable prognostic factor in colorectal cancer [51, 52]. This observation is consistent with the findings of our study. Neutrophils, another major component of immune infiltrates in rectal cancer, may exert complex and context‐dependent effects on tumor progression, with both tumor‐promoting and tumor‐suppressive functions reported in the literature [51, 53]. Previous studies have suggested that higher tumor‐associated neutrophil density is associated with improved response to 5‐fluorouracil‐based chemotherapy in colorectal cancer.

Our exploratory analyses further demonstrated that both neutrophil density and neutrophil proportion were associated with improved tumor response (Figures 4 and S2 and Table S4). Importantly, the prognostic impact of neutrophils may depend not only on their Ab but also on their spatial organization within the tumor microenvironment. Spatial analyses in the present study revealed that tumor‐infiltrating neutrophil Ab scores and the M–H index, which reflects immune–tumor cell colocalization, were significantly associated with favorable response to CRT (Figures 4 and S3 and Table S4).

Interestingly, the spatial relationship between lymphocytes and neutrophils also appeared to influence treatment response. Using the *G*_Lymphocyte:Neutrophil_ AUC_0,50 µm_ metric, which reflects the probability that lymphocytes have neutrophil neighbors within 50 µm, we found that higher *G*‐cross values were significantly associated with improved tumor response. These findings are consistent with those reported by Governa et al., who demonstrated that colocalization of tumor‐associated neutrophils with CD8^+^ T cells in colorectal cancer was associated with improved survival, potentially through neutrophil‐mediated enhancement of CD8^+^ T‐cell activation and cytokine production [54]. Similarly, Peng et al. reported that spatial proximity between T and B lymphocytes and N1‐like neutrophils promotes anti‐tumor immune responses and is associated with improved prognosis [55]. Collectively, these findings suggest that both immune cell composition and spatial architecture within the tumor microenvironment may influence response to CRT, highlighting the potential value of spatially resolved histopathological analysis for predicting treatment outcomes in LARC.

This study has several limitations. First, the single‐arm design lacked a control group, which limits direct comparison with other treatment strategies. Second, the sample size for the exploratory histopathological analyses was relatively small, and these findings should therefore be interpreted with caution. Third, although it was a multicenter trial, more than 85% of patients were enrolled at the primary study center, reflecting differences in institutional scale and patient volume across participating centers. Nevertheless, uniform treatment protocols were applied across all centers, and no statistically significant differences in outcomes were observed between the primary center and the other participating institutions, supporting the generalizability of our findings. Additionally, the COVID‐19 pandemic caused unavoidable delays in surgical scheduling for several patients, which may have influenced treatment timelines and outcomes.

In conclusion, the TESS trial demonstrates that the sandwich‐style TNT strategy achieves high rates of cCR, overall CR, and SP, with encouraging survival outcomes and manageable toxicity. This treatment approach may represent a safe and effective option for carefully selected patients with low‐risk distal LARC. By facilitating high cCR rates and enabling broader use of W&W, this strategy has the potential to increase organ preservation while maintaining favorable oncological outcomes and quality of life.

## 4 Materials and Methods

### 4.1 Study Design and Participants

The TESS trial (Total Neoadjuvant Treatment to Increase the Clinical Complete Response Rate for Distal Locally Advanced Rectal Cancer) was a prospective, open‐label, multicenter, single‐arm phase II study. The study protocol was registered at ClinicalTrials.gov (NCT03840239) [56], and the detailed study design and methodology have been reported previously [57]. The trial protocol and all subsequent amendments were approved by the Ethics Committee of Sun Yat‐Sen University Cancer Center (approval numbers: 5010‐2018‐04‐01 and 5010‐2018‐04‐03).

Eligible patients were 18–70 years of age with biopsy‐confirmed rectal adenocarcinoma staged as cT3‐4aNany or cT1‐4aN+, with the lower tumor margin located within 5 cm of the anal verge, as determined by pelvic MRI. Patients were required to have an Eastern Cooperative Oncology Group performance status of 0–1 and were assessed by colorectal surgeons as ineligible for or uncertain candidates for SPS. Patients with lateral lymph node metastasis or distant metastasis at diagnosis were excluded. Written informed consent was obtained from all participants prior to study enrollment and before initiation of any study‐related procedures.

### 4.2 Study Procedures and Assessments

Eligible patients received the sandwich TNT regimen (NeoCT‒CRT‒NeoCT), consisting of two cycles of neoadjuvant CapeOx chemotherapy administered before, during, and after radiotherapy. The chemotherapy regimen comprised six cycles of CapeOx. Oxaliplatin (130 mg/m^2^) was administered intravenously on day 1 of cycles 1, 2, 5, and 6, followed by capecitabine (1000 mg/m^2^) administered orally twice daily for 14 days, repeated every 3 weeks.

During cycles 3 and 4, oxaliplatin was administered at a reduced dose of 100 mg/m^2^ concurrent with radiotherapy. Radiotherapy was delivered in 25 fractions, consisting of 2.0 Gy per fraction (total dose 50.0 Gy) to the planning target volume (PTV) of the gross tumor volume (GTV) and nodal gross tumor volume (GTVnd), and 1.8 Gy per fraction (total dose 45.0 Gy) to the PTV of the clinical target volume (CTV).

Clinical tumor response was assessed 1 week after completion of neoadjuvant therapy. cCR was defined according to the 2020 Chinese Society of Clinical Oncology colorectal cancer guidelines, based on a comprehensive evaluation including digital rectal examination, endoscopy, and high‐resolution MRI. Tumor responses were categorized into three groups: cCR, near‐cCR, and non‐cCR. Detailed assessment criteria are provided in Table S6.

Patients who achieved cCR were recommended to undergo the W&W strategy. For patients with non‐cCR, surgical management was determined through multidisciplinary team discussion. All patients were advised to receive two cycles of adjuvant capecitabine therapy.

### 4.3 Follow‐Up Strategy

For patients who underwent surgery, follow‐up visits were scheduled every 3 months during the first 3 years, every 6 months between years 3 and 5, and annually thereafter. Follow‐up assessments included physical examination, digital rectal examination, tumor marker testing, and abdominal ultrasonography. Contrast‐enhanced pelvic MRI and contrast‐enhanced chest and abdominal CT were performed at least once annually, and PET‐CT was conducted when clinically indicated.

Patients managed with the W&W strategy underwent a more intensive surveillance protocol after confirmation of cCR. Follow‐up visits were scheduled every 3 months during the first 3 years, every 6 months between years 3 and 5, and annually thereafter. In addition to the routine assessments described above, each visit included colonoscopy and contrast‐enhanced pelvic MRI to facilitate early detection of local regrowth. Transrectal color Doppler ultrasound was performed when clinically indicated. Investigators were permitted to implement more frequent monitoring when considered clinically necessary. Any suspected local regrowth was managed promptly with salvage surgery or other appropriate treatment.

### 4.4 Endpoints

The primary endpoint was the cCR rate, evaluated with a minimum follow‐up of 1.5 years. The SP rate was defined as the proportion of patients who underwent SPS or avoided surgery. CR was defined as pCR after surgery or sustained cCR/near‐cCR for at least 1.5 years. Local regrowth was defined as tumor recurrence involving the rectal wall or regional lymph nodes after initial cCR/near‐cCR assessment with W&W, whereas local recurrence referred to rectal wall or regional lymph node recurrence after surgical treatment [39].

Secondary endpoints included pCR rate, surgical complications, adverse events, overall survival, disease‐free survival, distant‐metastasis‐free survival, and quality of life. Adverse events occurring during TNT were graded according to the Common Terminology Criteria for Adverse Events (CTCAE) (version V5.0).

### 4.5 Histopathological Factors

H&E‐stained WSIs were classified into nine tissue categories: background, adipose tissue, debris/necrosis, aggregated lymphocytes, mucus, smooth muscle, normal colonic mucosa, tumor‐associated stroma, and adenocarcinoma epithelium (representative images are shown in Figure S4) [34]. Based on this classification, tumor bulk regions were identified for subsequent analyses. The CoNIC challenge dataset was used as the training dataset for nuclear segmentation and annotation [35]. Cell detection and quantification were performed using the SFCN‐OPI framework [58].

To characterize tumor‐immune microenvironment features, multiple quantitative parameters describing immune cell Ab and spatial distribution were calculated, including *G*‐cross [59], immune cell surface density [60], tumor‐infiltrating cell Ab score, M‒H index [61], and stromal clustering/barrier metrics [62]. The *G*‐cross metric was calculated based on the probability distribution of immune cells located nearest to tumor cells within a predefined spatial distance. The immune cell Ab score integrates the immune cell‐to‐tumor cell ratio with colocalization statistics within tumor regions.

### 4.6 Statistical Analysis

Based on retrospective institutional data, the historical cCR rate following long‐course radiotherapy with concurrent capecitabine was estimated to be 17%. The study hypothesized that the sandwich TNT strategy would increase the cCR rate by 13 percentage points, from 17% to 30%. For the primary endpoint, a one‐sided proportion test and log‐rank test were used with a significance level of *α* = 0.025 and 80% statistical power. Assuming a 10% dropout rate, the required sample size was 98 patients, who were subsequently enrolled in the study.

The CR and SP rates were analyzed in both the ITT population and the observed population. The ITT population was defined as all enrolled patients who completed the protocol‐specified TNT regimen and underwent response assessment. In the ITT analysis, patients who did not achieve cCR after the standard TNT regimen but subsequently received a second course of radiotherapy were classified as non‐CR, regardless of their final response status.

The observed population included all patients who ultimately achieved CR, including those who attained cCR following a second course of radiotherapy administered at the physician's discretion. Baseline clinical characteristics were summarized for the overall cohort and by response group. Continuous variables were compared using mean differences, while categorical variables were analyzed using the chi‐square test or Fisher's exact test, as appropriate. Survival outcomes were estimated using the Kaplan‒Meier method, and differences between groups were evaluated using the long‐rank test.

Univariable logistic regression was performed to identify histopathological variables associated with treatment response, and variables meeting the selection criteria were subsequently included in multivariable logistic regression analysis. WSIs were analyzed using QuPath and a custom Python (version 3.11) pipeline. All statistical analyses were performed using R, and two‐sided *p*‐values <0.05 were considered statistically significant.

## Author Contributions

*Conception and design*: WeiWei Xiao, YuanHong Gao, and Gong Chen. *Protocol development*: Shuang Liu, GuangZhao Lv, XiaoZhong Wang, YeZhong Zhuang, ShouMin Bai, XiaoJun Wu, YiJing Ye, PeiQiang Cai, ZhiZhong Pan, YuanHong Gao, Gong Chen, and WeiWei Xiao. *Patient recruitment*: GuangZhao Lv, XiaoZhong Wang, YeZhong Zhuang, ShouMin Bai, XiaoJun Wu, YiJing Ye, ZhiZhong Pan, QiaoXuan Wang, Hui Chang, YuanHong Gao, Gong Chen, and WeiWei Xiao. *Data collection, analysis, and interpretation*: Shuang Liu, GuangZhao Lv, GeYu Xu, HuiLong Luo, HaiNa Yu, and WeiWei Xiao. *Manuscript drafting and revision*: Shuang Liu, GuangZhao Lv, and WeiWei Xiao. All the authors had full access to the study data, participated in the review and revision of the manuscript, and approved the final version prior to submission.

## Funding

This work was supported by the 5010 Clinical Research Foundation of Sun Yat‐sen University Clinical Research 5010 Program (grant number: 2018030).

## Ethics Statement

This study was registered with clinicaltrials.gov (NCT03840239). The study protocol and all amendments were approved by the Ethics Committee of Sun Yat‐sen University Cancer Center (approval numbers: 5010‐2018‐04‐01 and 5010‐2018‐04‐03). All procedures involving human participants were conducted in accordance with the ethical standards of the institutional research committee and the Declaration of Helsinki. Written informed consent was obtained from all participants prior to enrollment in the study.

## Conflicts of Interest

Author ShuoYu Xu is an employee in Bio‐Totem Pte Ltd, but has no potential relevant financial or non‐financial interests to disclose. The other authors have no conflicts of interest to declare.

## Supporting information




**Supporting File 1**: mco270807‐sup‐0001‐SuppMat.docx

## Data Availability

The datasets generated and/or analyzed during the current study are available from the corresponding author upon reasonable request.
