# Supplementary material for: Safety and Efficacy of a Sandwich Total Neoadjuvant Therapy Strategy for Low‐Risk Distal Locally Advanced Rectal Cancer: Results From the TESS Phase II Trial
Source: MedComm (2020). 2026 Jun 17;7(7):e70807. doi: 10.1002/mco2.70807 (PMC13273842; doi:10.1002/mco2.70807)
Supplement: Supplementary file 1 — Supporting File 1: mco270807‐sup‐0001‐SuppMat.docx [file MCO2-7-e70807-s001.docx]

**Safety and efficacy of a sandwich total neoadjuvant therapy strategy for low-risk distal locally advanced rectal cancer: results from the TESS phase II trial**

Shuang Liu^1-4,#^, GuangZhao Lv^2,5,#^, GeYu Xu^2,6,#^, XiaoZhong Wang^7,#^, YeZhong Zhuang^8,#^, ShouMin Bai^9^ , HaiNa Yu^1,2,10^, XiaoJun Wu^2,5^, YiJing Ye^11^, HuiLong Luo^1,2^, ShuoYu Xu^12^, QiaoXuan Wang^1,2^, Hui Chang^1,2^, PeiQiang Cai^2,6^, ZhiZhong Pan^2,5^, YuanHong Gao^1,2,^*, Gong Chen^2,5,^*, WeiWei Xiao^1,2,13^*

**Affiliations**

^1^ Department of Radiation Oncology, Sun Yat-sen University Cancer Center, Guangzhou, China

^2^ Sun Yat-sen University Cancer Center, State Key Laboratory of Oncology in South China, Collaborative Innovation Center for Cancer Medicine, Guangzhou, China

^3^ Department of Hematology, Oncology, and Tumor Immunology and Berlin Institute of Health, Charité-Universitätsmedizin Berlin, Germany

^4^ Berlin Institute for Medical Systems Biology (BIMSB), Max Delbrück Center for Molecular Medicine, Berlin, Germany

^5^ Department of Colorectal Surgery, Sun Yat-sen University Cancer Center, Guangzhou, China

^6^ Department of Medical Imaging and Interventional Radiology, Sun Yat-sen University Cancer Center, Guangzhou, China

^7^ Department of General Surgery, Shantou Central Hospital, Shantou, China

^8^ Department of Abdominal Surgery, Cancer Hospital of Shantou University Medical College, Shantou, China

^9^ Department of Radiation Oncology, Sun Yat-sen Memorial Hospital, Sun Yat-sen University, Guangzhou, China

^10^ Department of Medical Oncology, Jinjiang Municipal Hospital (Shanghai Sixth People's Hospital Fujian), Jinjiang, China

^11^ Department of Radiation Oncology, Zhongshan People's Hospital, Zhongshan, China

^12^ Bio-totem Pte Ltd, Suzhou, China

^13^ United Laboratory of Frontier Radiotherapy Technology of Sun Yat-sen University & Chinese Academy of Sciences Ion Medical Technology Co., Ltd, Guangzhou, China

^#^These authors contributed equally to this work.

*Corresponding authors:

YuanHong Gao, MD

Department of Radiation Oncology, Sun Yat-sen University Cancer Center, Guangzhou 510060, China Tel: +86-20-87343385

Fax: +86-20-87343385

Email: gaohy@sysucc.org.cn

Gong Chen, MD

Department of Colorectal Surgery, Sun Yat-sen University Cancer Center, Guangzhou 510060, China

Tel: +86-20-87343584

Fax: +86-20-87343584

Email: chengong@sysucc.org.cn

WeiWei Xiao, MD, PhD (Lead Corresponding Author)

Department of Radiation Oncology, Sun Yat-sen University Cancer Center, Guangzhou 510060, China Tel: +86-20-87343491

Fax: +86-20-87343491

Email: xiaoww@sysucc.org.cn

**Supplementary Information**

**Supplementary Tables**

Table S1. Comparison of baseline clinical characteristics and treatment between CR and non-CR patients in the intention-to-treat (ITT) population

Table S2. Comparison of baseline clinical characteristics and treatment between CR and non-CR patients in the observed response population

Table S3. Comparison of baseline clinical characteristics and treatment between CR and non-CR patients in the exploratory histopathological analysis cohort

Table S4. Univariate analysis of pretreatment histopathological features associated with clinical response (CR vs non-CR) in the exploratory histopathological analysis cohort

Table S5. Comparative outcomes of total neoadjuvant therapy (TNT) regimens for locally advanced rectal cancer

Table S6. Clinical complete response (cCR) assessment criteria and representative images

**Supplementary Figures**

Figure S1. Treatment and outcomes of patients who underwent a second course of radiotherapy

Figure S2. Comparison of immune cell density and proportion between non-CR and CR patients

Figure S3. Spatial distribution analysis of tumor cells and immune cells between non-CR and CR patients

Figure S4. Representative H&E-stained whole-slide images and corresponding tissue classification

**Table S1. Comparison of baseline clinical characteristics and treatment between CR and non-CR patients in the intention-to-treat (ITT) population**

| **Variable** | **No.** | **Non-CR (%)** | **CR (%)** | **P value** |
| --- | --- | --- | --- | --- |
| All patients | 98 | 41 (42%) | 57 (58%) |  |
| Age |  |  |  | 0.97 |
| < 55 | 44 | 19 (43%) | 25 (57%) |  |
| ≥ 55 | 54 | 22 (41%) | 32 (59%) |  |
| Sex |  |  |  | 0.458 |
| Female | 34 | 12 (35%) | 22 (65%) |  |
| Male | 64 | 29 (45%) | 35 (55%) |  |
| Distance to the anal verge (cm) |  |  |  | 0.561 |
| < 3.5 | 50 | 19 (38%) | 31 (62%) |  |
| ≥ 3.5 | 48 | 22 (46%) | 26 (54%) |  |
| CEA (ng/ml) |  |  |  | 0.463 |
| < 5 | 65 | 25 (39%) | 40 (61%) |  |
| ≥ 5 | 33 | 16 (49%) | 17 (51%) |  |
| CA19-9 (U/ml) |  |  |  | 0.373 |
| < 35 | 88 | 35 (40%) | 53 (60%) |  |
| ≥ 35 | 10 | 6 (60%) | 4 (40%) |  |
| T stage |  |  |  | 0.622 |
| T2 | 6 | 2 (33%) | 4 (67%) |  |
| T3 | 81 | 33 (41%) | 48 (59%) |  |
| T4a | 11 | 6 (55%) | 5 (45%) |  |
| N stage |  |  |  | 0.462 |
| N0 | 28 | 9 (32%) | 19 (68%) |  |
| N1 | 43 | 20 (47%) | 23 (53%) |  |
| N2 | 27 | 12 (44%) | 15 (56%) |  |
| MRF |  |  |  | 0.782 |
| Negative | 53 | 21 (40%) | 32 (60%) |  |
| Positive | 45 | 20 (44%) | 25 (56%) |  |
| EMVI |  |  |  | 0.672 |
| Negative | 75 | 30 (40%) | 45 (60%) |  |
| Positive | 23 | 11 (48%) | 12 (52%) |  |
| Cycles of neoadjuvant chemotherapy |  |  |  | 0.133 |
| 4 | 4 | 3 (75%) | 1 (25%) |  |
| 5 | 7 | 5 (71%) | 2 (29%) |  |
| 6 | 80 | 30 (38%) | 50 (62%) |  |
| 7 | 5 | 3 (60%) | 2 (40%) |  |
| 8 | 2 | 0 (0%) | 2 (100%) |  |
| Cycles of adjuvant chemotherapy |  |  |  | 0.48 |
| 2 | 95 | 39 (41%) | 56 (59%) |  |
| 3 | 1 | 1 (100%) | 0 (0%) |  |
| 4 | 2 | 1 (50%) | 1 (50%) |  |

Abbreviations: MRF, mesorectal fascia; EMVI, extramural venous invasion; CEA, carcinoembryonic antigen; CA19-9, carbohydrate antigen 19-9. Values are presented as number (%). Percentages represent the proportion within each row.

**Table S2. Comparison of baseline clinical characteristics and treatment between CR and non-CR patients in the observed response population**

| **Variable** | **No.** | **Non-CR (%)** | **CR (%)** | **P value** |
| --- | --- | --- | --- | --- |
| All patients | 98 | 38 (39%) | 60 (61%) |  |
| Age |  |  |  | 0.855 |
| < 55 | 44 | 18 (41%) | 26 (59%) |  |
| ≥ 55 | 54 | 20 (37%) | 34 (63%) |  |
| Sex |  |  |  | 0.766 |
| Female | 34 | 12 (35%) | 22 (65%) |  |
| Male | 64 | 26 (41%) | 38 (59%) |  |
| Distance to the anal verge (cm) |  |  |  | 0.434 |
| < 3.5 | 50 | 17 (34%) | 33 (66%) |  |
| ≥ 3.5 | 48 | 21 (44%) | 27 (56%) |  |
| CEA (ng/ml) |  |  |  | 0.455 |
| < 5 | 65 | 23 (35%) | 42 (65%) |  |
| ≥ 5 | 33 | 15 (46%) | 18 (54%) |  |
| CA19-9 (U/ml) |  |  |  | 0.67 |
| < 35 | 88 | 33 (38%) | 55 (62%) |  |
| ≥ 35 | 10 | 5 (50%) | 5 (50%) |  |
| T stage |  |  |  | 0.514 |
| T2 | 6 | 2 (33%) | 4 (67%) |  |
| T3 | 81 | 30 (37%) | 51 (63%) |  |
| T4a | 11 | 6 (55%) | 5 (45%) |  |
| N stage |  |  |  | 0.182 |
| N0 | 28 | 7 (25%) | 21 (75%) |  |
| N1 | 43 | 18 (42%) | 25 (58%) |  |
| N2 | 27 | 13 (48%) | 14 (52%) |  |
| MRF |  |  |  | 0.983 |
| Negative | 53 | 20 (38%) | 33 (62%) |  |
| Positive | 45 | 18 (40%) | 27 (60%) |  |
| EMVI |  |  |  | 0.776 |
| Negative | 75 | 28 (37%) | 47 (63%) |  |
| Positive | 23 | 10 (43%) | 13 (57%) |  |
| Cycles of neoadjuvant chemotherapy |  |  |  | 0.321 |
| 4 | 4 | 3 (75%) | 1 (25%) |  |
| 5 | 7 | 4 (57%) | 3 (43%) |  |
| 6 | 80 | 29 (36%) | 51 (64%) |  |
| 7 | 5 | 2 (40%) | 3 (60%) |  |
| 8 | 2 | 0 (0%) | 2 (100%) |  |
| Cycles of adjuvant chemotherapy |  |  |  | 0.424 |
| 2 | 95 | 36 (38%) | 59 (62%) |  |
| 3 | 1 | 1 (100%) | 0 (0%) |  |
| 4 | 2 | 1 (50%) | 1 (50%) |  |

Abbreviations: MRF, mesorectal fascia; EMVI, extramural venous invasion; CEA, carcinoembryonic antigen; CA19-9, carbohydrate antigen 19-9. Values are presented as number (%). Percentages represent the proportion within each row.

**Table S3. Comparison of baseline clinical characteristics and treatment between CR and non-CR patients in the exploratory histopathological analysis cohort**

| **Variable** | **No.** | **Non-CR (%)** | **CR (%)** | **P value** |
| --- | --- | --- | --- | --- |
| All patients | 50 | 20 (40%) | 30 (60%) |  |
| Age |  |  |  | 0.953 |
| < 55 | 21 | 9 (43%) | 12 (57%) |  |
| ≥ 55 | 29 | 11 (38%) | 18 (62%) |  |
| Sex |  |  |  | 0.266 |
| Female | 21 | 6 (29%) | 15 (71%) |  |
| Male | 29 | 14 (48%) | 15 (52%) |  |
| Distance to the anal verge (cm) |  |  |  | 0.953 |
| < 3.5 | 29 | 11 (38%) | 18 (62%) |  |
| ≥ 3.5 | 21 | 9 (43%) | 12 (57%) |  |
| CEA (ng/ml) |  |  |  | 0.345 |
| < 5 | 35 | 12 (34%) | 23 (66%) |  |
| ≥ 5 | 15 | 8 (53%) | 7 (47%) |  |
| CA19-9 (U/ml) |  |  |  | 0.328 |
| < 35 | 44 | 16 (36%) | 28 (64%) |  |
| ≥ 35 | 6 | 4 (67%) | 2 (33%) |  |
| T stage |  |  |  | 0.336 |
| T2 | 2 | 1 (50%) | 1 (50%) |  |
| T3 | 42 | 15 (36%) | 27 (64%) |  |
| T4a | 6 | 4 (67%) | 2 (33%) |  |
| N stage |  |  |  | 0.328 |
| N0 | 12 | 3 (25%) | 9 (75%) |  |
| N1 | 24 | 12 (50%) | 12 (50%) |  |
| N2 | 14 | 5 (36%) | 9 (64%) |  |
| MRF |  |  |  | 1 |
| Negative | 24 | 10 (42%) | 14 (58%) |  |
| Positive | 26 | 10 (39%) | 16 (61%) |  |
| EMVI |  |  |  | 0.153 |
| Negative | 41 | 14 (34%) | 27 (66%) |  |
| Positive | 9 | 6 (67%) | 3 (33%) |  |
| Cycles of neoadjuvant chemotherapy |  |  |  | 0.125 |
| 4 | 3 | 3 (100%) | 0 (0%) |  |
| 5 | 1 | 1 (100%) | 0 (0%) |  |
| 6 | 43 | 15 (35%) | 28 (65%) |  |
| 7 | 2 | 1 (50%) | 1 (50%) |  |
| 8 | 1 | 0 (0%) | 1 (100%) |  |
| Cycles of adjuvant chemotherapy |  |  |  | 0.21 |
| 2 | 1 | 1 (100%) | 0 (0%) |  |
| 3 | 48 | 18 (38%) | 30 (62%) |  |
| 4 | 1 | 1 (100%) | 0 (0%) |  |

Abbreviations: MRF, mesorectal fascia; EMVI, extramural venous invasion; CEA, carcinoembryonic antigen; CA19-9, carbohydrate antigen 19-9. Values are presented as number (%). Percentages represent the proportion within each row.

**Table S4. Univariate analysis of pretreatment histopathological features associated with clinical response (CR vs non-CR) in the exploratory histopathological analysis cohort**

| Variable | Non-CR (N=20) | CR (N=30) | OR [95%CI] | P value | |
| --- | --- | --- | --- | --- | --- |
| Tumor bulk proportion in total tissue area | 0.44 (0.25) | 0.44 (0.22) | 1.08 [0.09;13.2] | 0.951 | |
| Tumor area proportion in total tissue area | 0.23 (0.15) | 0.20 (0.13) | 0.25 [0.00;14.6] | 0.502 | |
| Lymphocyte area proportion in total tissue area | 0.05 [0.02;0.08] | 0.06 [0.04;0.09] | 949 [0.00; Inf] | 0.338 | |
| Mucus area proportion in total tissue area | 0.02 [0.01;0.05] | 0.03 [0.01;0.05] | 7066 [0.00; Inf] | 0.33 | |
| Stromal area proportion in total tissue area | 0.07 [0.02;0.17] | 0.06 [0.03;0.12] | 0.48 [0.00;101] | 0.789 | |
| Ratio of stromal area to total stromal and tumor areas | 0.29 [0.09;0.56] | 0.25 [0.14;0.53] | 1.21 [0.17;8.47] | 0.849 | |
| Ratio of mucus area to total mucus and tumor areas | 0.12 [0.03;0.25] | 0.13 [0.07;0.23] | 2.05 [0.20;20.6] | 0.541 | |
| Stromal area proportion in the tumor bulk | 0.07 [0.03;0.20] | 0.10 [0.05;0.13] | 2.53 [0.02;390] | 0.718 | |
| Tumor area proportion in the tumor bulk | 0.54 [0.37;0.58] | 0.42 [0.31;0.54] | 0.07 [0.00;3.49] | 0.18 | |
| Lymphocyte area proportion in the tumor bulk | 0.09 [0.04;0.12] | 0.07 [0.05;0.12] | 1.99 [0.00;6825] | 0.869 | |
| Mucus area proportion in the tumor bulk | 0.02 [0.01;0.03] | 0.02 [0.01;0.05] | 21.1 [0.00;151536] | 0.501 | |
| Cell density in the tumor region of the tumor bulk | | | | |  |
| Lymphocyte | 6.63 [6.10; 6.88] | 6.83 [6.29; 7.15] | 1.31 [0.61; 2.79] | 0.484 | |
| Neutrophil | 3.51 (0.79) | 4.14 (0.94) | 2.34 [1.12; 4.90] | 0.024 | |
| Plasma cell | 4.59 (0.79) | 4.91 (0.86) | 1.58 [0.79; 3.17] | 0.195 | |
| Stromal cell | 7.40 [7.23; 7.63] | 7.51 [7.26; 7.61] | 0.96 [0.25; 3.65] | 0.953 | |
| Cell proportion in the tumor bulk | | | | |  |
| Lymphocyte | -1.98 (0.49) | -1.71 (0.41) | 4.25 [1.01; 17.9] | 0.049 | |
| Neutrophil | -5.09 (0.85) | -4.50 (0.93) | 2.15 [1.04; 4.45] | 0.038 | |
| Plasma cell | -3.71 [-4.21; -3.36] | -3.41 [-3.85; -3.04] | 1.85 [0.82; 4.20] | 0.141 | |
| Stromal cell | -1.15 (0.33) | -1.03 (0.27) | 4.28 [0.57; 32.3] | 0.158 | |
| Cell-to-tumor cell ratio in the tumor bulk | | | | |  |
| Lymphocyte | -1.21 (0.66) | -0.65 (0.71) | 3.63 [1.30; 10.1] | 0.014 | |
| Neutrophil | -4.32 (0.85) | -3.44 (1.08) | 2.63 [1.26; 5.51] | 0.01 | |
| Plasma cell | -3.11 (0.77) | -2.51 (0.93) | 2.30 [1.09; 4.86] | 0.029 | |
| Stromal cell | -0.38 (0.55) | 0.03 (0.60) | 3.63 [1.16; 11.4] | 0.027 | |
| G _Tumor:Cell_ AUC_0,50μm_ quartiles in the tumor bulk | | | | |  |
| Lymphocyte | 3.24 [3.12; 3.31] | 3.24 [3.13; 3.40] | 1.04 [0.15; 7.33] | 0.968 | |
| Neutrophil | 1.02 (0.77) | 1.41 (0.94) | 1.69 [0.86; 3.33] | 0.125 | |
| Plasma cell | 2.35 [1.72; 2.49] | 2.40 [1.80; 2.75] | 1.23 [0.54; 2.82] | 0.622 | |
| Stromal cell | 3.45 [3.41; 3.48] | 3.43 [3.38; 3.46] | 0.01 [0.00; 18.4] | 0.241 | |
| M-H index of cell in the tumor bulk | | | | |  |
| Lymphocyte | -1.02 (0.31) | -0.89 (0.25) | 5.73 [0.64; 50.9] | 0.117 | |
| Neutrophil | -3.26 (0.80) | -2.54 (0.78) | 3.36 [1.37; 8.26] | 0.008 | |
| Plasma cell | -2.08 [-2.43; -1.76] | -1.58 [-2.15; -1.36] | 2.03 [0.91; 4.49] | 0.083 | |
| Stromal cell | -0.65 (0.18) | -0.71 (0.23) | 0.26 [0.02; 4.44] | 0.352 | |
| Tumor infiltrating cell Ab score in the tumor bulk | | | | |  |
| Lymphocyte | -2.64 (0.88) | -2.05 (0.68) | 2.83 [1.20; 6.67] | 0.017 | |
| Neutrophil | -7.44 (1.72) | -6.01 (1.71) | 1.67 [1.12; 2.48] | 0.012 | |
| Plasma cell | -5.26 (1.58) | -4.36 (1.74) | 1.39 [0.96; 1.99] | 0.079 | |
| Stromal cell | -1.46 (0.58) | -1.16 (0.46) | 3.24 [0.98; 10.8] | 0.055 | |
| Cell density in the tissue region |  |  |  |  | |
| Lymphocyte | 759 (380) | 940 (453) | 1.00 [1.00; 1.00] | 0.15 | |
| Neutrophil | 32.9 [6.74; 56.9] | 42.0 [17.5; 107] | 1.01 [1.00; 1.03] | 0.062 | |
| Plasma cell | 127 [77.8; 181] | 156 [96.0; 242] | 1.00 [1.00; 1.01] | 0.133 | |
| Stromal cell | 1651 [1207; 1938] | 1614 [1338; 2070] | 1.00 [1.00; 1.00] | 0.325 | |
| Cell proportion in the tissue region | | | |  | |
| Lymphocyte | -1.75 (0.34) | -1.61 (0.31) | 4.18 [0.65; 27.1] | 0.133 | |
| Neutrophil | -5.13 (0.81) | -4.69 (0.99) | 1.71 [0.89; 3.31] | 0.109 | |
| Plasma cell | -3.36 [-3.88; -3.11] | -3.33 [-3.78; -2.86] | 1.52 [0.61; 3.80] | 0.368 | |
| Stromal cell | -0.99 (0.35) | -0.94 (0.33) | 1.60 [0.29; 8.89] | 0.588 | |
| Cell-to-tumor cell ratio in the tissue region | | | | | |
| Lymphocyte | -0.69 (0.69) | -0.33 (0.66) | 2.35 [0.90; 6.11] | 0.08 | |
| Neutrophil | -4.07 (0.84) | -3.41 (1.11) | 1.98 [1.04; 3.77] | 0.038 | |
| Plasma cell | -2.49 (0.77) | -2.12 (0.76) | 1.97 [0.88; 4.41] | 0.1 | |
| Stromal cell | 0.08 (0.77) | 0.34 (0.81) | 1.56 [0.73; 3.34] | 0.254 | |
| Cell density in the tumor bulk |  |  |  |  | |
| Lymphocyte | 786 [642; 1009] | 973 [707; 1474] | 1.00 [1.00; 1.00] | 0.196 | |
| Neutrophil | 44.1 [22.4; 65.2] | 68.0 [23.0; 111] | 1.01 [1.00; 1.03] | 0.058 | |
| Plasma cell | 156 [78.8; 189] | 189 [92.6; 292] | 1.01 [1.00; 1.01] | 0.071 | |
| Stromal cell | 1906 (558) | 2117 (791) | 1.00 [1.00; 1.00] | 0.303 | |
| Cell density in the stromal region of the tumor bulk | | | | | |
| Lymphocyte | 1502 [945; 2325] | 1919 [1282; 2821] | 1.00 [1.00; 1.00] | 0.634 | |
| Neutrophil | 64.1 [40.9; 91.7] | 87.8 [38.3; 159] | 1.01 [1.00; 1.02] | 0.106 | |
| Plasma cell | 210 [106; 404] | 351 [161; 521] | 1.00 [1.00; 1.00] | 0.142 | |
| Stromal cell | 3099 (630) | 3449 (839) | 1.00 [1.00; 1.00] | 0.123 | |
| Cell density in the stromal region |  |  |  |  | |
| Lymphocyte | 1436 [876; 2523] | 2015 [1292; 2685] | 1.00 [1.00; 1.00] | 0.655 | |
| Neutrophil | 59.9 [12.4; 82.6] | 72.9 [26.2; 163] | 1.01 [1.00; 1.01] | 0.109 | |
| Plasma cell | 208 [145; 397] | 351 [158; 496] | 1.00 [1.00; 1.00] | 0.173 | |
| Stromal cell | 3035 (874) | 3458 (852) | 1.00 [1.00; 1.00] | 0.103 | |
| G _Neutrophil:Lymphocyte_ AUC_0,50μm_ quartiles in the tumor bulk | 2.64 [2.16;2.89] | 2.89 [2.42;3.19] | 2.95 [1.02;8.53] | 0.046 | |

Abbreviations:∞: infinity; OR: odds ratio.

Continuous variables are presented as mean (SD) or median (IQR), as appropriate.

**Table S5. Comparative outcomes of total neoadjuvant therapy (TNT) regimens for locally advanced rectal cancer**

| Study | Patients | Primary endpoint | Arm | Treatment strategy details | cCR | pCR | cCR with local regrowth | Grade ≥3 toxicities | LRR | DFS | DMFS | OS |
| --- | --- | --- | --- | --- | --- | --- | --- | --- | --- | --- | --- | --- |
| POLISH II | cT4 or fixed cT3 | R0 | TNT | 5×5Gy -> FOLFOX * 3 -> Surgery | / | 16% | / | 24% | 35% | 43% | 64% | 49% |
|  |  |  | Standard | CRT [50.4 Gy + 5-FU/ OXA] -> Surgery | / | 12% | / | 24% | 32% | 41% | 66% | 49% |
| RAPIDO | cT4a or cT4b, extramural vascular invasion, cN2, involved mesorectal fascia, or enlarged lateral lymph nodes | DrTF | TNT | 5 × 5 Gy -> CapeOx * 6 or FOLFOX * 9 ‐> Surgery | / | 28% | / | 48% | 17% | 23.7% | 67% | 89.1% |
|  |  |  | Standard | CRT [28 × 1.8Gy or 25 × 2Gy + Cape] -> Surgery -> CapeOx * 8 or FOLFOX * 12 | / | 14% | / | 25% | 10% | 30.4% | 81% | 88.8% |
| PRODIGE-23 | rectal adenocarcinoma within 15 cm of the anal verge, stage cT3 (at risk of LR and for which a multidisciplinary tumor board recommended preoperative CRT) or cT4. | DFS | TNT | mFOLFIRINOX * 6 -> CRT [50.4Gy/25F + Cape] -> Surgery -> mFOLFOX6 * 6 or Cape * 4 | / | 27.8% | / | 45% | 19.5% | 76% | 79% | 91% |
|  |  |  | Standard | CRT [50.4Gy/25F + Cape] – TME ‐ mFOLFOX * 12 or Cape * 8 | / | 12.10% | / | 76% | 27% | 69% | 72% | 88% |
| OPRA | cT3–T4N0, or any T, N1-2 | DFS | INCT-CRT | INCT [mFOLFOX * 8 or CapeOx * 5] -> CRT [54 Gy + 5-FU or Cape] -> Surgery or W&W | 71% | 75% | 40% | 41% | 6% | 76% | 84% | 90.5% |
|  |  |  | CRT-CNCT | CRT [54 Gy + 5-FU or Cape] -> CNCT [mFOLFOX * 8 or CapeOx * 5]-> Surgery or W&W | 76% | 78% | 27% | 34% | 6% | 76% | 82% | 92.8% |
| CAO/ARO/AIO-12 | rectal adenocarcinoma up to 12 cm above the anal verge; cT3 tumor less than 6 cm from the anal verge, cT3 cancer in the middle third of the rectum (≥6–12 cm) with extramural tumor spread into the mesorectum of more than 5 mm (>cT3b), cT4 tumors, or lymph node involvement. | pCR | INCT-CRT | FOLFOX * 3 -> CRT [50.4 Gy + FU/ OXA] -> Surgery | / | 17.00% | / | 37% | 6% | 73% | 82% | 92% |
|  |  |  | CRT-CNCT | CRT [50.4 Gy + 5-FU/ OXA] -> FOLFOX * 3 -> Surgery | / | 25.00% | / | 27% | 5% | 73% | 84% | 92% |
| TNTCRT | LARC patients with high risk factors of recurrence: cT4a-b (resectable) cT3c-d with EMVI+ cN2 MRF+ | DFS | TNT | CapeOx * 1-> CRT [50-50.4 Gy / 25-28 F + CapeOx * 2] -> CapeOx * 3 | 13/232 | 27.51% | / | 28.02% | 2.59% | 77.0% | 83.0% | 90.30% |
|  |  |  | Standard | CRT [50-50.4 Gy / 25-28 F + Cape] -> Surgery -> CapeOx or Cape | 2/222 | 9.84% | / | 22.07% | 3.54% | 67.9% | 74.2% | 87.9% |
| STELLAR | distal or middle-third, clinical primary tumor stage 3-4 and/or regional lymph node–positive rectal cancer | DFS | TNT | 5 × 5Gy -> CapeOx * 4 -> Surgery -> CapeOx * 2 | 11.1% | 17.2% | 7.1% | 26.5% | 8.40% | 64.5% | 77.1% | 86.5% |
|  |  |  | Standard | CRT [50 Gy / 25 F + Cape] -> Surgery -> CapeOx * 6 | 4.4% | 13.9% | 10.0% | 12.6% | 11.00% | 62.3% | 75.3% | 75.1% |
| TESS | cT3-4 Nany or cT1-4aN+, tumor located less than 5cm from the anal verge, sphincter-preserving surgery is not applicable or not sure by colorectal surgeon’s evaluation, without lateral lymph node metastasis or distant metastasis | cCR | TNT | CapeOx * 2 -> CRT [50.4Gy/25F + CapeOx * 2] -> CapeOx * 2 -> Surgery or W&W | 46.9% | 46.4% | 9.7% | 27.6% | 0.0% | / | 92.9% | 96.9% |

Abbreviations: TNT, total neoadjuvant therapy; CRT, chemoradiotherapy; TME, total mesorectal excision; LARC, locally advanced rectal cancer; pCR, pathological complete response; cCR, clinical complete response; LRR, local relapse rate; DFS, disease-free survival; DMFS, distant metastasis-free survival; OS, overall survival; W&W, watch-and-wait; DrTF, disease-related treatment failure; INCT-CRT, induction chemotherapy followed by CRT; CRT-CNCT, CRT followed by consolidation chemotherapy; Cape, capecitabine; CapeOx, capecitabine plus oxaliplatin; FOLFOX, folinic acid, fluorouracil, and oxaliplatin; 5-FU, fluorouracil; OXA, oxaliplatin; FOLFIRINOX, folinic acid, fluorouracil, irinotecan, and oxaliplatin; MRF, mesorectal fascia; EMVI, extramural venous invasion.

**Table S6. Clinical complete response (cCR) assessment criteria and representative images**

|  | cCR | Near-cCR | Non-cCR |
| --- | --- | --- | --- |
| Digital rectal examination (DRE) | Normal | Minor mucosal abnormality | Palpable tumor nodules |
| Endoscopic criteria | White and flat mucosal scars | Irregular rectal mucosa or other minor mucosal abnormalities, including scarring with mild persistent mucosal erythema, shallow ulcers, or small nodules | Visible tumor |
| High-resolution MRI criteria | T2WI: Normal appearing rectal wall or homogeneous low-signal fibrosis at the primary tumor site, without intermediate signal intensity.  DWI: No high signal intensity on high b-values* at the primary tumor site.  ADC: No low-signal intensity areas suggestive of residual tumor, with only linear fibrotic signal in the tumor bed.  No suspicious lymph nodes present. | T2WI: Predominantly low-signal fibrotic scar with minimal residual intermediate signal intensity, and partial reduction in lymph node size.  DWI: Mild or equivocal hyperintensity on high b-values*, with significant signal regression from baseline.  ADC: Minimal residual low-signal intensity.  Residual lymph nodes may be present but appear morphologically benign without diffusion restriction. | T2WI: Persistent intermediate or heterogeneous signal intensity at the primary tumor site, with mass-like nodularity.  DWI: Clearly hyperintense signal on high b-value*.  ADC: Persistent low-signal areas present in the area corresponding to the DWI hyperintensity.  Suspicious lymph nodes present. |
| Representative endoscopic images | Baseline  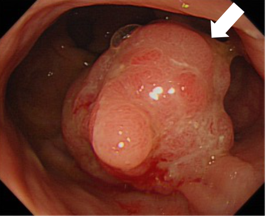  After TNT  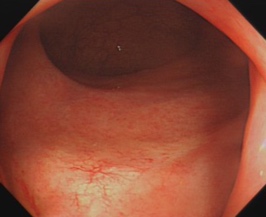  Baseline endoscopy revealed a raised mass occupying the majority of the rectal lumen (white arrow).  After TNT, follow-up endoscopy revealed mild mucosal swelling in the lower rectum with no obvious mass identified. | Baseline  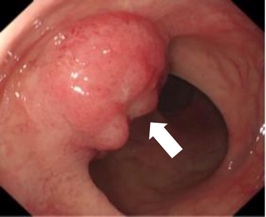  After TNT  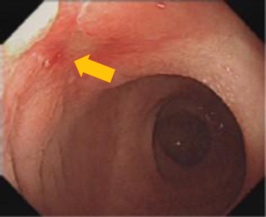  Baseline endoscopy revealed an exophytic, protruding mass located 4–5 cm from the anal verge, growing into the lumen (white arrow).  After TNT, follow-up endoscopy revealed a well-healed ulcer scar located 4-5 cm from the anal verge, with central fibrinous exudate and no evidence of residual mass (yellow arrow). | Baseline  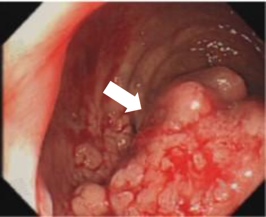  After TNT  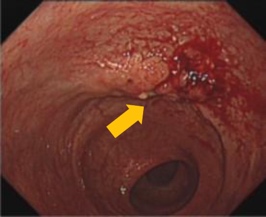  Baseline endoscopy revealed a cauliflower-like mass in the lower rectum, approximately 2–5 cm from the anal verge (white arrow).  After TNT, follow-up endoscopy revealed persistent mucosal congestion with nodular surface elevation in the lower rectum, approximately 3–5 cm from the anal verge (yellow arrow). |
| MRI T2WI representative images | Baseline  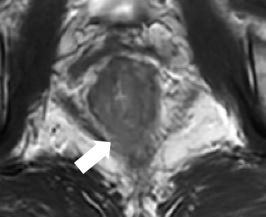  After TNT  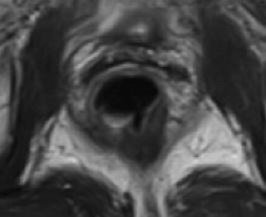  Baseline MRI demonstrated intermediate signal intensity on T2WI (white arrow), with tumor extension beyond the muscularis propria into the perirectal fat.  After TNT, post-therapy MRI demonstrated only fibrotic signal (dark T2 signal) at the primary tumor site, with no residual intermediate signal intensity. | Baseline  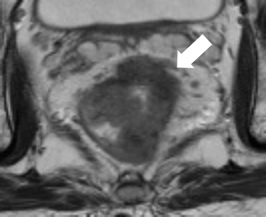  After TNT  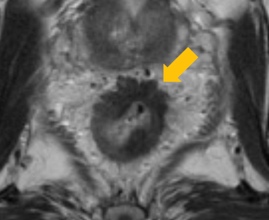  Baseline MRI demonstrated the tumor as an intermediate T2WI lesion, with extramural extension into the perirectal fat (white arrow).  After TNT, post-therapy MRI demonstrated a fibrotic scar with minimal residual intermediate signal intensity at the primary tumor site (yellow arrow). | Baseline  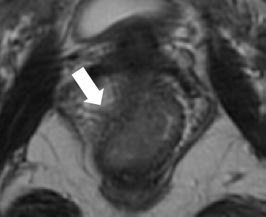  After TNT  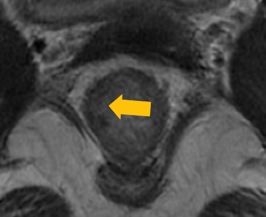  Baseline MRI demonstrated intermediate signal intensity on T2WI, with tumor extension beyond the muscularis propria into the perirectal fat (white arrow).  After TNT, post-therapy MRI demonstrated intermediate signal intensity breaching the inner hypointense layer, with substantial residual intermediate signal intensity (yellow arrow). |
| MRI DWI representative images | Baseline  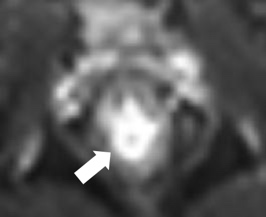  After TNT  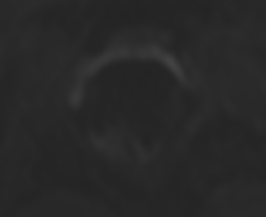  Baseline MRI demonstrated high signal intensity on DWI (white arrow).  After TNT, post-therapy MRI demonstrated complete resolution of the previously noted high signal intensity on DWI. | Baseline  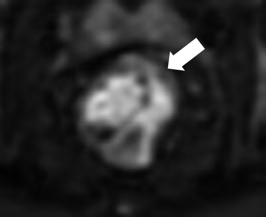  After TNT  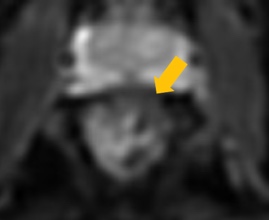  Baseline MRI demonstrated high signal intensity on DWI (white arrow).  After TNT, post-therapy MRI demonstrated few punctate foci of mildly high signal intensity on DWI (yellow arrow). | Baseline  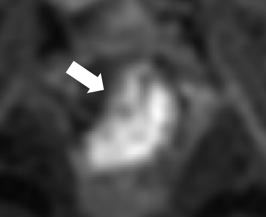  After TNT  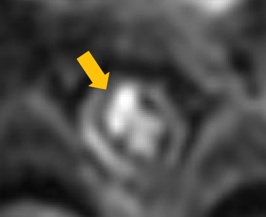  Baseline MRI demonstrated high signal intensity on DWI (white arrow).  After TNT, post-therapy MRI demonstrated persistent focal high signal intensity on DWI (yellow arrow). |
| MRI ADC representative images | Baseline  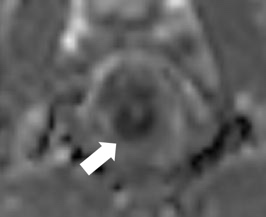  After TNT  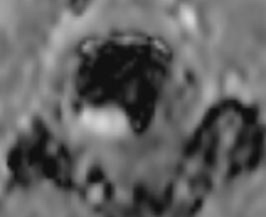  Baseline MRI demonstrated low signal intensity on ADC map (white arrow).  After TNT, post-therapy MRI demonstrated complete resolution of the previously noted high signal intensity on ADC map. | Baseline  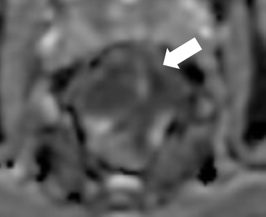  After TNT  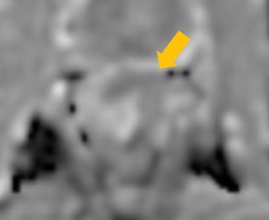  Baseline MRI demonstrated low signal intensity on ADC (white arrow).  After TNT, post-therapy MRI demonstrated persistently mild low signal intensity on the corresponding ADC map (yellow arrow). | Baseline  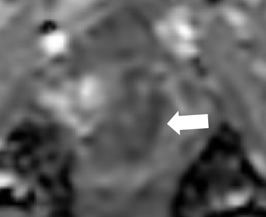  After TNT  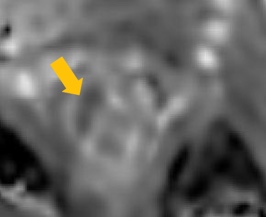  Baseline MRI demonstrated low signal intensity on ADC (white arrow).  After TNT, post-therapy MRI demonstrated persistent focal low signal intensity on the corresponding ADC map (yellow arrow). |

*: b ≥ 800 sec/mm².

Abbreviations: TNT, total neoadjuvant treatment; T2WI, T2-weighted imaging; DWI, diffusion-weighted imaging; ADC, apparent diffusion coefficient.


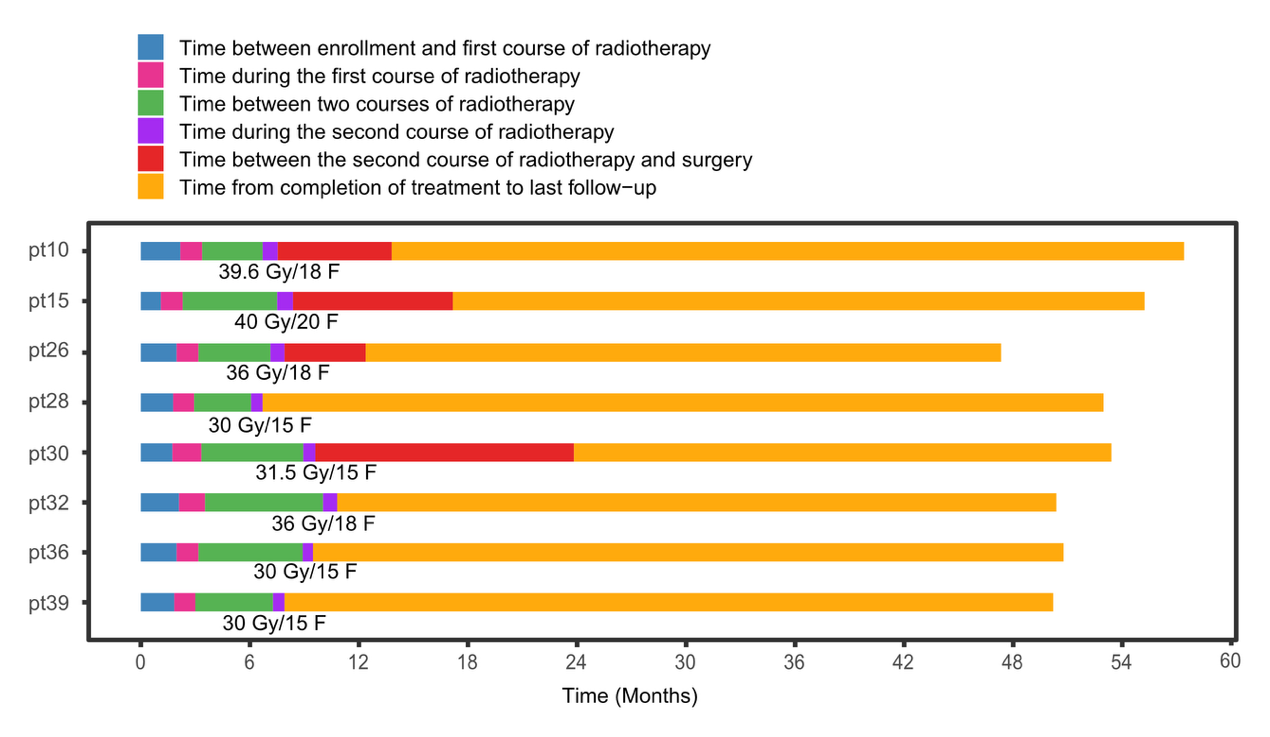


**Figure S1. Treatment and outcomes of patients who underwent a second course of radiotherapy.** One patient (pt36) was evaluated as near-cCR, and the other seven patients were evaluated as non-cCR at the first assessment. Four patients (pt28, pt30, pt32, and pt39) received a second course of radiotherapy and subsequently achieved cCR. One patient (pt30) underwent sphincter-preserving surgery (SPS). Three patients (pt10, pt15, pt26) underwent non-sphincter-preserving surgery. One patient (pt26) achieved pCR, while three patients (pt10, pt15, and pt30) did not achieve pCR. Abbreviations: near-cCR, near-complete clinical response; non-cCR, non-complete clinical response; pCR, pathological complete response; SPS, sphincter-preserving surgery.


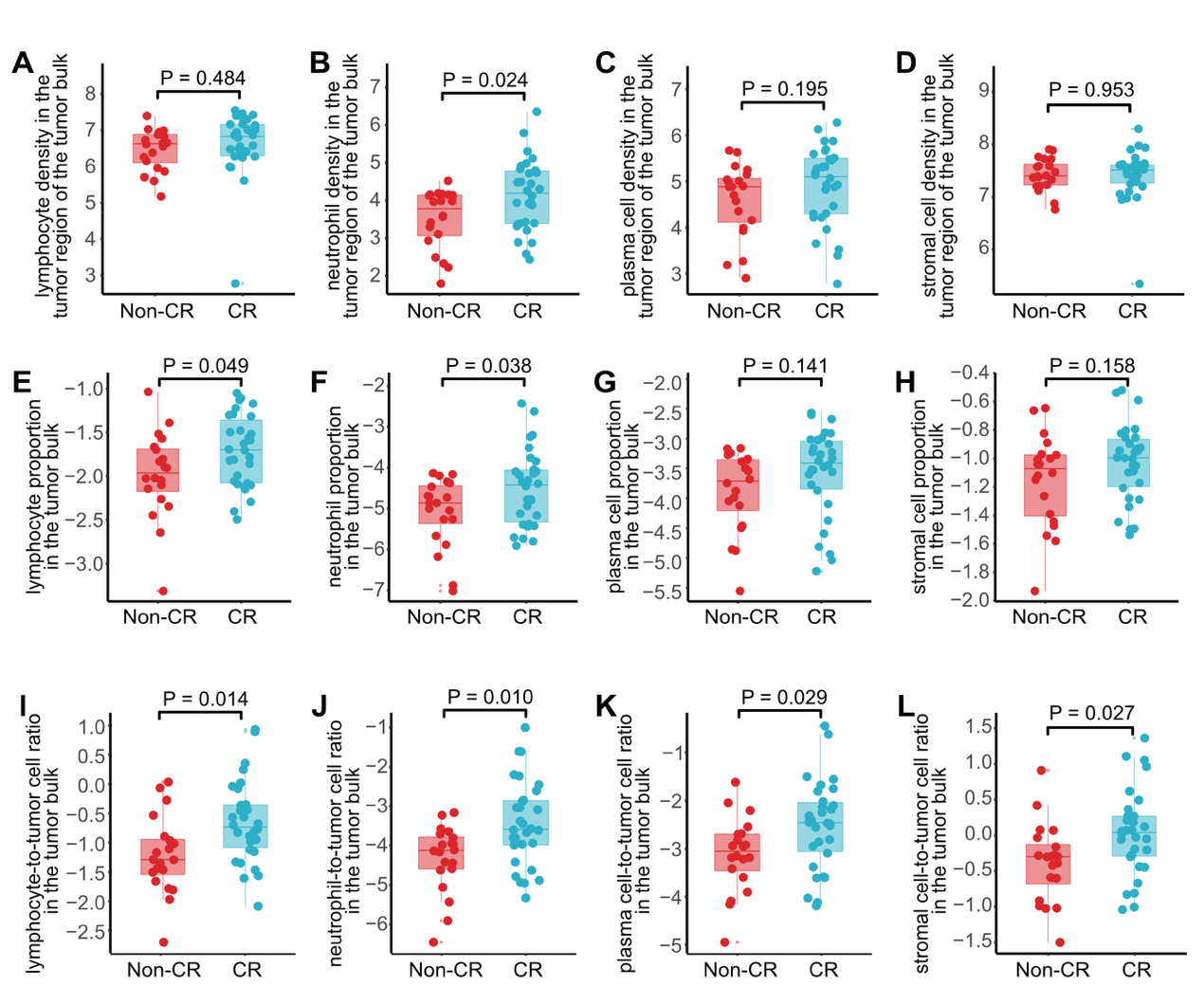


**Figure S2. Comparison of immune cell density and proportion between non-CR and CR patients.**

**(**A-D) Boxplots showing the density of lymphocytes (A), neutrophils (B), plasma cells (C), and stromal cells (D) in the tumor region of the tumor bulk.

(E-H) Boxplots showing the proportion of lymphocytes (E), neutrophils (F), plasma cells (G), and stromal cells (H) in the tumor bulk.

(I-L) Boxplots showing the cell-to-tumor cell ratios for lymphocytes (I), neutrophils (J), plasma cells (K), and stromal cells (L) in the tumor bulk.

Data were log-transformed prior to analysis to improve normality.


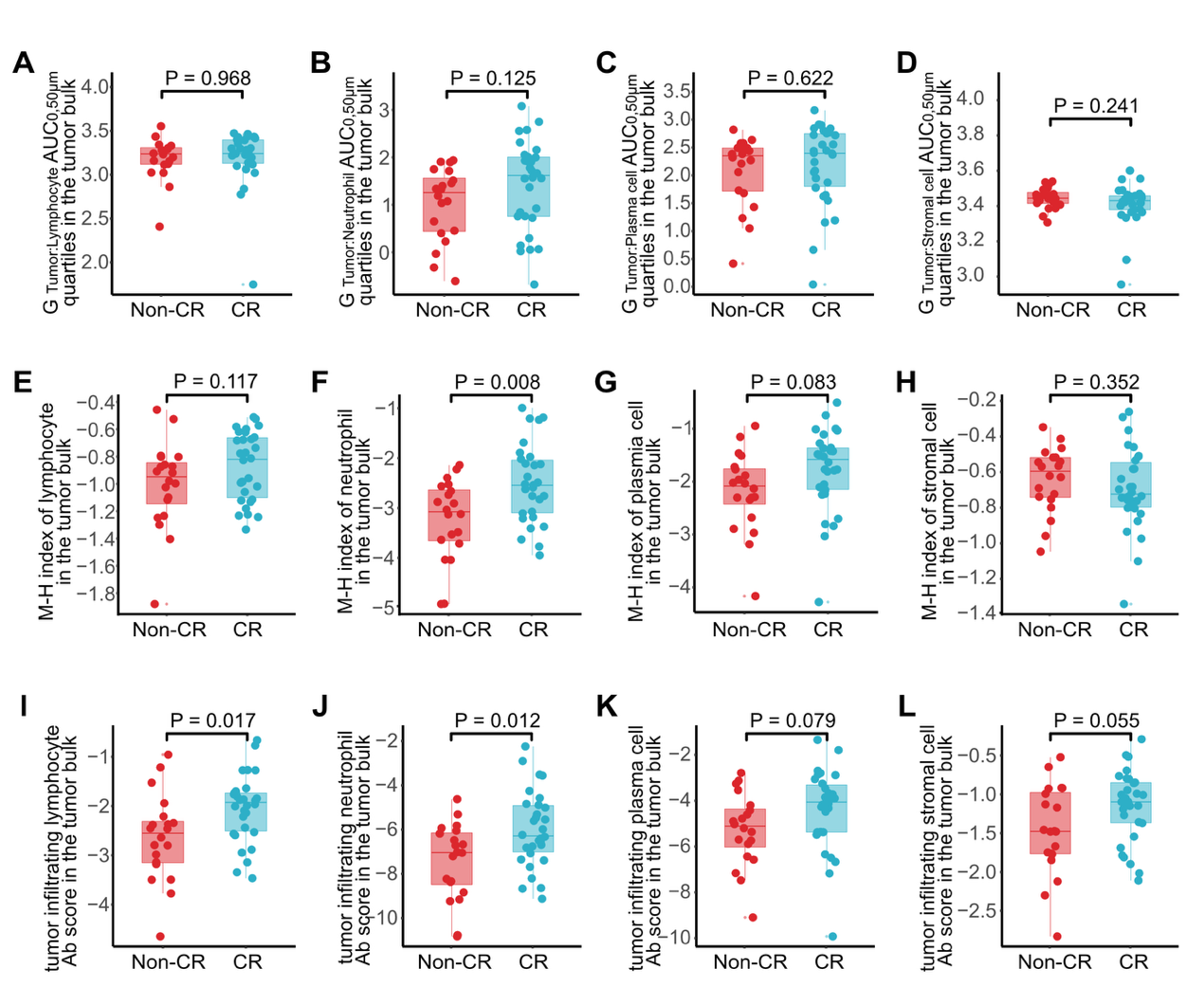


**Figure S3. Spatial distribution analysis of tumor cells and immune cells between non-CR and CR patients.**

**(**A–D) Boxplots showing G_Tumor:lymphocyte_ (A), neutrophil (B), plasma cell (C), and stromal cell (D) AUC_0, 50 μm_ quartiles in the tumor bulk.

(E–H) Boxplots showing the Morisita-Horn index (M-H index) for lymphocytes (E), neutrophils (F), plasma cells (G), and stromal cells (H) in the tumor bulk.

(I-L) Boxplots showing the tumor-infiltrating cell abundance (Ab) score for lymphocytes (I), neutrophils (J), plasma cells (K), and stromal cells (L) in the tumor bulk.

G _Tumor: specific cell_ AUC_0, 50 μm_, estimates the probability that a tumor cell has at least one neighboring cell of the specified type within a 50 μm radius.

Data were log-transformed prior to analysis to improve normality.

*
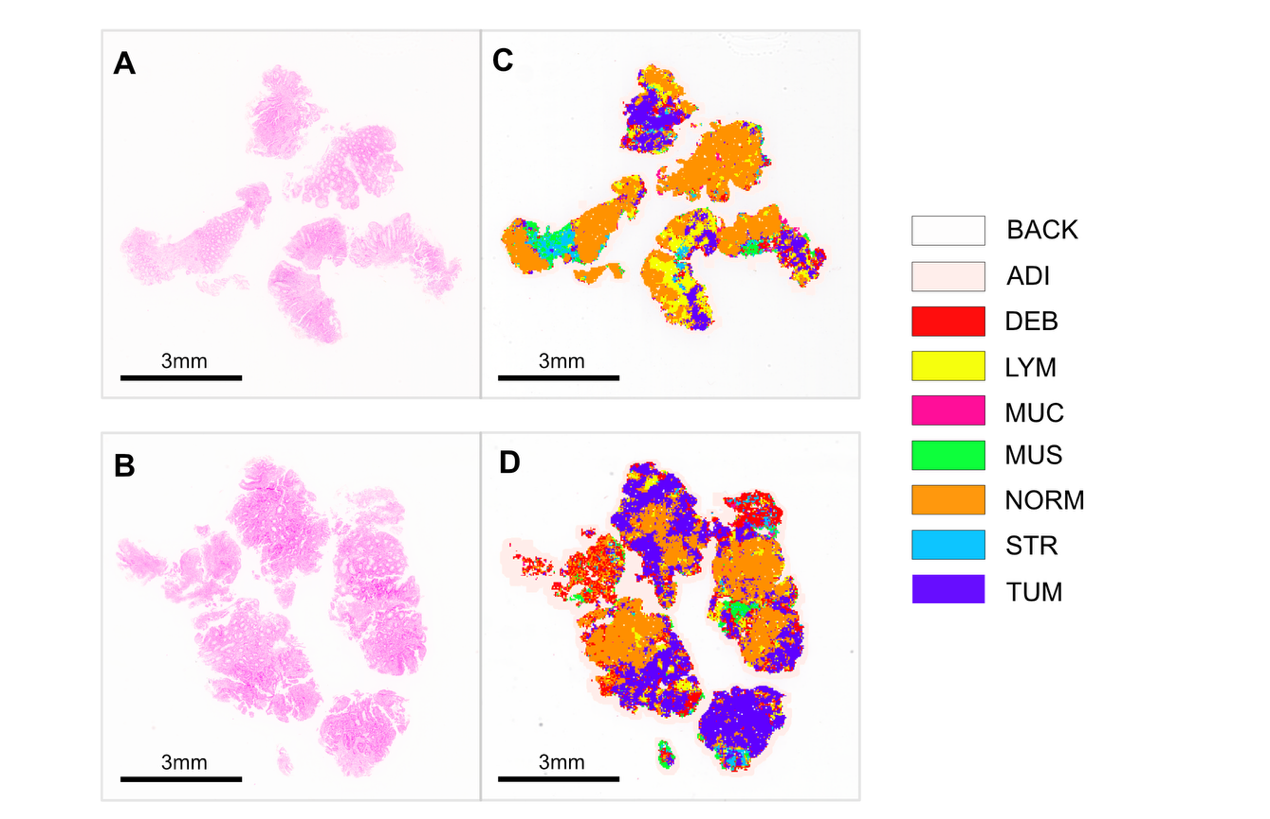
*

**Figure S4. Representative H&E-stained whole-slide images and corresponding tissue classification.** Panels A and B show representative H&E-stained whole-slide images. Panels C and D show the corresponding tissue classification into nine tissue types, with each tissue type annotated in distinct colors: BACK, background; ADI, adipose tissue; DEB, debris/necrosis; LYM, aggregated lymphocytes; MUC, mucus; MUS, smooth muscle; NORM, normal colon mucosa; STR, tumor-associated stroma; and

TUM, adenocarcinoma epithelium. Scale bars indicate 3 mm.
